# The STAR score: a method for auditing clinical records

**DOI:** 10.1308/003588412X13171221499865

**Published:** 2012-05

**Authors:** H Tuffaha, T Amer, P Jayia, C Bicknell, N Rajaretnam, P Ziprin

**Affiliations:** St Mary’s Hospital, LondonUK

**Keywords:** Note keeping, CRABEL, Audit, Structured admission forms, Scoring tool

## Abstract

**INTRODUCTION:**

Adequate medical note keeping is critical in delivering high quality healthcare. However, there are few robust tools available for the auditing of notes. The aim of this paper was to describe the design, validation and implementation of a novel scoring tool to objectively assess surgical notes.

**METHODS:**

An initial ‘path finding’ study was performed to evaluate the quality of note keeping using the CRABEL scoring tool. The findings prompted the development of the Surgical Tool for Auditing Records (STAR) as an alternative. STAR was validated using inter-rater reliability analysis. An audit cycle of surgical notes using STAR was performed. The results were analysed and a structured form for the completion of surgical notes was introduced to see if the quality improved in the next audit cycle using STAR. An education exercise was conducted and all participants said the exercise would change their practice, with 25% implementing major changes.

**RESULTS:**

Statistical analysis of STAR showed that it is reliable (Cronbach’s a = 0.959). On completing the audit cycle, there was an overall increase in the STAR score from 83.344% to 97.675% (p<0.001) with significant improvements in the documentation of the initial clerking from 59.0% to 96.5% (p<0.001) and subsequent entries from 78.4% to 96.1% (p<0.001).

**CONCLUSIONS:**

The authors believe in the value of STAR as an effective, reliable and reproducible tool. Coupled with the application of structured forms to note keeping, it can significantly improve the quality of surgical documentation and can be implemented universally.

In 1995 the Audit Commission maintained that ‘proper management of health records and accurate, comprehensive record-keeping are essential to effective patient care and continuity of care between different health professionals’. This assertion was made as part of a wide-ranging report on hospital health records, *Setting the Record Straight.^1^* Our study had two aims: to improve case note availability within hospitals and to ensure the quality and reliability of their contents.

An agreed case note structure was recommended and guidelines for adhering to such standards were disseminated. Included within these guidelines were good practice principles covering legibility, patient identification, diagnosis, treatment, nursing records, diagnostic tests, housekeeping of notes and ensuring confidentiality. It was stipulated that regular review of performance against those standards should be undertaken. One such review in 1999 found that the basic standards of record keeping had significantly improved by 8% in four years.^2^ Despite a national improvement in most standards, there is still considerable variation between trusts in their improvements to the structure and contents of patients’ records.^2^

Adequate medical note keeping is essential across all medical and surgical specialties. It is often determined to be inadequate and causal in adverse events at medicolegal reviews.^3^^–^^5^ However, the methods of auditing these notes vary between hospitals. Current methods and tools are usually considered to be too extensive and tedious to be performed on a regular basis. Crawford *et al* developed the CRABEL (CRAwford – BEresford – Lafferty) scoring system to assess the quality of clinical note keeping.^6^ Using this system, a numerical score is assigned to specific components of a case note series that are regarded as significant in medical note keeping. This system has been previously validated as an audit tool.^7^ Crawford *et al’&* intention was to facilitate the audit of note keeping between clinical teams and to enable it to be applied to any inpatient specialty, medical or surgical.

The aim of this paper was to describe the design, validation and implementation of a novel scoring tool to objectively assess surgical notes. This is, in brief, a modification of the CRABEL tool and more emphasis is placed on the quality of clinical documentation.

## Methods

### Pilot study

An initial ‘path finding’ assessment was performed to evaluate the quality of note keeping using the CRABEL scoring tool. The note assessment was performed as described in the original paper.^6^ The sample size was increased to 20 consecutive case notes to assess for conformity of results. The CRABEL tool was subjected to a thorough qualitative examination by an expert panel. The results were critiqued in correlation with the actual findings in the notes, the weighting of different sections in the tool and the reliability of the results.

### STAR design

The Surgical Tool for Auditing Records (STAR) was developed using Royal College of Surgeons (RCS) guidelines on medical record keeping.^8^ It consists of 50 essential entries that should exist in any complete set of surgical notes. The chosen entries aimed to accurately reflect documented guidance with regards to medical notes. Most of the guidance was incorporated into the design of the tool except for sections relating to postmortem reports, nursing plans and intensive care unit admission as these were deemed to be separate entities in case notes. The weighted sections incorporated into scores were: admission (20%), subsequent entries (16%), consent (14%), anaesthetic record (14%), operative record (18%) and discharge summary (18%)

This weighting ensures that scores appropriately reflect the state of note keeping in the document as a whole, thus limiting the scope for error that would arise if sections were weighted differently. The division of STAR into sections was also thought to be beneficial as it enables subgroup analysis of results to look for specific deficiencies and areas for improvement.

### STAR score

**Figure 1:**
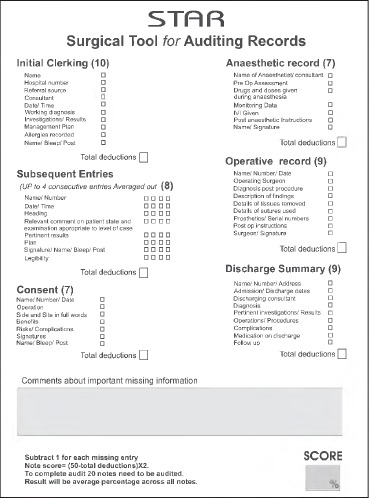
STAR scoring sheet

The scoring template is divided to reflect documentation of the patient case notes as described above. These divisions are then subdivided into essential elements that render the entry complete. Points are deducted for each missing component (Fig 1).

*Initial clerking (10 points):* This should be the first entry of a member of the surgical team. Essential components include: (i) patient name at the top of the clerking section; (ii) patient hospital number; (iii) referral source (GP, A&E, ...); (iv) consultant in charge; (v) date and time the patient was seen; (vi) working diagnosis; (vii) investigations ordered and results available; (viii) initial management plan; (ix) allergies; and (x) name, post and bleep number of the person creating the entry. Each entry should be complete and a point is deducted if not.

*Subsequent entries (8 points):* Up to four consecutive entries can be examined. Entries should contain: (i) patient name and hospital number on the continuation sheet; (ii) date and time of the entry; (iii) an entry heading; (iv) a comment about observations or the general state of the patient (ie well, unwell, ...) that is appropriate for the category of the patient; (v) pertinent results; (vi) a management plan; (vii) signature, name, bleep number and post; and (viii) the entries should be legible (the presence of two illegible words renders the entry illegible). When calculating this subscore, total deductions should be divided by the number of entries examined to maintain uniformity in scoring. For example, if 4 entries are examined and 20 points need to be deducted, then the actual points deducted for this are 5.

*Consent (7 points):* A point is deducted for each of the following if missing: (i) patient name, hospital number and the date; (ii) operation performed; (iii) site and side in full words; (iv) benefits of the intended procedure; (v) risks and complications; (vi) signatures of the patient and the consenting doctor; and (vii) name, post and bleep number of the consenting doctor.

*Anaesthetic record (7 points):* This should be examined for: (i) name of anaesthetist; (ii) preoperative assessment; (iii) drugs and doses given during anaesthesia; (iv) presence of monitoring data; (v) intravenous fluids given during anaesthesia; (vi) postanaesthetic instructions; and (vii) name and signature of the person making the entry.

*Operative record (9 points):* A point is deducted for each missing entry of the following: (i) patient name, hospital number and date of operation; (ii) name of the operating surgeon; (iii) diagnosis after the procedure; (iv) description of operative findings; (v) details of tissues removed; (vi) details of sutures used; (vii) prosthetics with serial numbers if used; (viii) postoperative instructions; and (ix) surgeon’s name and signature.

*Discharge summary (9 points):* The entries looked at include: (i) patient name, hospital number and address; (ii) admission and discharge dates; (iii) discharging consultant; (iv) diagnosis at discharge; (v) pertinent investigations and results; (vi) operations and procedures undertaken; (vii) presence or absence of complications; (viii) medications on discharge; and (ix) follow up.

### STAR methodology

Twenty consecutive sets of notes are examined on discharge. The notes are reviewed and scored according to the STAR proforma (Fig 1). Points are deducted for each missing entry and, on completion of the task, the total number of deductions in the case note should be calculated. Having analysed the whole case note series, a STAR score can be calculated by using the formula:

1,000 – total deductions in the series / 10

The number 1,000 is derived from 20 (sets of notes) x 50 (points per case note) and represents a perfect score.

### STAR validation

STAR was validated using inter-rater reliability analysis. Seven different doctors independently assessed the same set of four notes using STAR (ie a total of 28 note reviews). The rater scores were then compared and analysed to calculate Cronbach’s a and the mean inter-item correlation value.

### Audit cycle

An audit was performed using STAR. As described, 20 consecutive case notes were audited of patients who were just discharged from the vascular unit at St Mary’s Hospital, London. The same auditor assessed all notes in the sample. The auditor was involved in the design of the tool and therefore did not require training. Scores and results were compiled and then analysed, looking at the overall score and for possible deficiencies in certain areas of note keeping. Remedial changes in the form of structured notes were introduced to assess improvement. A re-audit was performed following the changes using the same methods as above. Pre-and post-audit scores were compared. The difference between the single case note scores and scores of individual sections of pre-and post-audit sample populations were analysed for statistical significance using the Mann-Whitney U test.

### Assessment of the educational value of STAR

STAR was assessed for its educational value by asking twenty doctors and students to complete a structured questionnaire assessing perceived educational value before and after auditing one set of notes. The pre-exercise question naires included previous note auditing experience, awareness of note keeping standards and subjective assessments of their note keeping standard. Post-exercise questions asked included whether the exercise was thought to be useful, whether it gave the subject insight into his or her own documentation standards and whether the exercise would change his or her practice. The final questions in the pre-and post-exercise questionnaires asked the subjects if they believed that junior doctors’ induction should include training on medical note keeping.

## Results

### Pilot study

Examination of the CRABEL proforma revealed several issues:

1. The score relied heavily on technical/procedural aspects of note keeping such as patient name and hospital number while giving less weight to important content such as patient medical status and allergies.
2. Anaesthetic records and operation notes were not examined in CRABEL.
3. The CRABEL score is unequally weighted (20% initial clerking, 60% subsequent entries, 10% consent, 10% discharge summary), which creates a score bias towards subsequent entries. This can potentially cause the score to not accurately reflect the overall status of the notes.
4. A sample size of two case notes was thought to be inadequate as this cannot reflect the status of note keeping in a whole department. In the pilot assessment, the CRABEL score of the 20 case notes put together was 90.2%; when scored according to the original description (two notes at a time) the results varied widely, ranging between 82% and 96% (Table 1).
5. Using STAR, the same case notes scored 90.5%, which is very close to their CRABEL score of 90.2%. The noticeable difference between the tools lies in the percentage of missing information picked up in each section, which was higher in STAR (Table 2).
6. STAR did not take longer to carry out; the number of points scored was the same as for the CRABEL scoring tool.
  For the aforementioned reasons, the department adopted STAR as the tool of preference to perform this audit.

**Table 1:** CRABEL score for each set of two notes inaseries of 20 case notes

Set	1	2	3	4	5	6	7	8	9	10
Score	96%	86%	89%	86%	87%	93%	94%	91%	94%	82%

**Table 2:** Omissions detected in each section of notes using the same 20 case notes

	CRABEL	STAR
Initial clerking	33/200 (16.5%)	41/200 (20.5%)
Subsequent entries	54/600 (9.0%)	21.5/160 (13.4%)
Consent	5/100 (5.0%)	8/140 (5.7%)
Operative record	–	4/180 (2.2%)
Anaesthetic record	–	8/140 (5.7%)
Discharge summary	6/100 (6.0%)	17/180 (9.4%)

### Design and validation

Statistical analysis of STAR showed that it is reliable and reproducible between observers with a Cronbach’s a of 0.959. The mean inter-item correlation score was 0.820.

### Audit cycle

In the initial audit, 166.56 points were deducted in total giving an overall STAR score of 83.34%. The median score was 85.5% with a range of 68.5% to 94.0%. Of all the deductions, 70% (116.56/166.56) were for the initial clerking (49.2%) and subsequent entry (20.8%) sections. The other sections contributed the remaining 30% of the deductions (Tables 3).

**Table 3:** Deductions from each individual section showing the percentage of missing information

Field	Total deductions / maximum points	Percentage of missing information
Initial clerking	82/200	41%
Subsequent entries	34.56/160	22%
Consent	3/140	2%
Operative record	9/180	5%
Anaesthetic record	22/140	16%
Discharge summary	16/180	9%
**Total deductions**	**166.56/1,000**	**17%**

Detailed analysis of the initial clerking showed that poor documentation of the referral source (12.2%), consultant in charge (18.3%), date and time (13.4%), and the name/bleep number/post of the person making the entry (15.8%) were the factors largely contributing to deductions from this section. The same applied to subsequent entries with the patient name and hospital number (26.8%), date and time (23.4%), legibility (13.4%) and details of the person making the entry (16.4%) making the most significant contributions.

**Figure 2:**
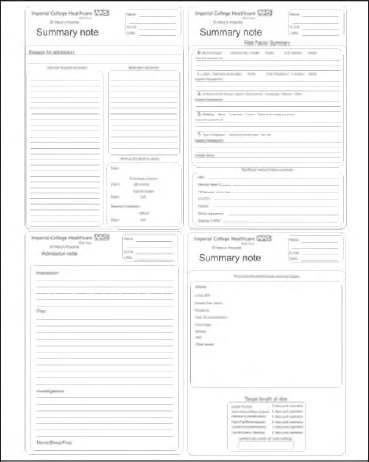
Sample of structured notes

It was found that the notes being used at that time lacked a structured format for both the admission clerkings and the subsequent entries compared to other sections of the notes. These shared the common attributes of having a specific proforma and template. Remedial actions were taken including the education of doctors and allied health professionals, and separating old and new notes to enable easy access. Most importantly, new proformas were designed for initial clerking and subsequent entries (Fig 2).

On completing the audit cycle, there was an overall increase in the STAR score from 83.344% to 97.675% (pcO.OOl) with significant improvements in the documentation of the initial clerking and subsequent entries sections (Table 4). There were also improvements in the documentation of other sections but these were not statistically significant.

**Table 4:** Differences in scores between the pre-and post-change audits

Field	Pre-change deductions	Post-change deductions	*p*-value (Mann–Whitney U test)
Initial clerking	82	7	<0.001
Subsequent entries	34.56	6.25	<0.001
Consent	3	1	0.602
Operative record	9	0	0.277
Anaesthetic record	22	8	0.060
Discharge summary	16	1	0.052
**Total deductions**	**166.56**	**23.25**	<0.001
**Score**	**83%**	**98%**	**<0.001**

### Teaching value

Twenty doctors and students underwent the exercise. Only three had audited notes before and six people were aware of the existence of note keeping standards. Of the 20 exercise participants, 50% thought the note keeping standard was adequate, 20% thought it was inadequate and 30% did not know. All of those questioned found the exercise useful, with 40% finding the exercise very useful. Nineteen subjects said that it gave them insight into their own documentation standards. AH the participants said the exercise would change their practice, with 25% implementing major changes. The number of people believing that junior doctors’ induction should include training on medical note keeping went up from 15 to 19 after the exercise.

## Discussion

The RCS produced specific guidelines for the maintenance of medical notes to be used as a standard for note keeping.^8^ Based on this standard and following the recommendations of *Setting the Record Straight,^1^* the authors of the CRABEL paper believe their tool to be a quick, easy and reproducible method for assessing and evaluating how closely the guidelines produced by the RCS are adhered to.^6^ They suggest that whereas previous audit tools assess the content of medical notes, the CRABEL score audits the quality of the actual medical record keeping.

Numerous auditors who have published their findings based on CRABEL share this view. Ho *et al* suggest that systemic audit cycles using CRABEL can lead to improvements in the note keeping process to the benefit of patient care.^9^ Dhariwal and Gibbons believe it is a ‘successful and objective measure for auditing and improving the quality of note-keeping’.^7^

However, while the CRABEL score has been recognised as a possible standard tool for auditing surgical notes, our own experience of CRABEL suggests there is scope for improvement. We believe STAR is an improvement on the CRABEL tool because it not only incorporates the expected aspects of initial clerking, subsequent entries, consent forms and discharge letters but also components that are critical in all surgical case notes, eg the anaesthetic record and the operative record. Details such as allergy and patient status are also relevant in medical notes and should be included in the overall assessment. These added variables and the larger audit sample make STAR a more evenly distributed and accurate measure of the quality of surgical note keeping throughout the surgical inpatient journey.

The use of structured admission and continuation sheets is still widely variable between different units^1^^–^^2^ even though evidence and sentiments lean more towards forms. The Royal College of Physicians conducted a national survey that confirmed that a significant number of clinicians believe that the same standardised headings should be used in the proforma for acute medical admissions in all NHS hospitals.^10^ Studies comparing structured case notes against free text notes concluded that structured forms were superior and are not onerous on time to complete.^11^^–^^14^ The superiority of adding structure to entries has been clearly demonstrated in our study, adding more evidence to the case of standardising note structure across all hospitals.

The educational value of STAR has not been statistically assessed in our study but the assessment performed showed that it has the potential to be beneficial in educating junior doctors in the areas of documentation and note keeping.

## Conclusions

We believe that STAR has value as an effective, reliable and reproducible auditing tool. Coupled with the application of structured forms to note keeping, it can significantly improve the quality of documentation and can be implemented universally. This study also highlights the potential benefit of using STAR as an educational adjunct. However, a further prospective study is required to confirm this assertion.
